# Locking the 150-Cavity Open: *In Silico* Design and Verification of Influenza Neuraminidase Inhibitors

**DOI:** 10.1371/journal.pone.0073344

**Published:** 2013-08-27

**Authors:** Nanyu Han, Yuguang Mu

**Affiliations:** School of Biological Sciences, Nanyang Technological University, Singapore, Singapore; University of Akron, United States of America

## Abstract

Neuraminidase (NA) of influenza is a key target for virus infection control and the recently discovered open 150-cavity in group-1 NA provides new opportunity for novel inhibitors design. In this study, we used a combination of theoretical methods including fragment docking, molecular linking and molecular dynamics simulations to design ligands that specifically target at the 150-cavity. Through *in silico* screening of a fragment compound library on the open 150-cavity of NA, a few best scored fragment compounds were selected to link with Zanamivir, one NA-targeting drug. The resultant new ligands may bind both the active site and the 150-cavity of NA simultaneously. Extensive molecular dynamics simulations in explicit solvent were applied to validate the binding between NA and the designed ligands. Moreover, two control systems, a positive control using Zanamivir and a negative control using a low-affinity ligand 3-(p-tolyl) allyl-Neu5Ac2en (ETT, abbreviation reported in the PDB) found in a recent experimental work, were employed to calibrate the simulation method. During the simulations, ETT was observed to detach from NA, on the contrary, both Zanamivir and our designed ligand bind NA firmly. Our study provides a prospective way to design novel inhibitors for controlling the spread of influenza virus.

## Introduction

Influenza A viruses infect a wide range of avian and mammalian hosts. The worldwide spread of avian flu as well as the subsequent outbreak of the 2009 H1N1 flu has raised public concerns of the global influenza pandemics due to the high morbidity and mortality [1,2,3]. Vaccines and antiviral drugs are two available strategies in preventing and controlling influenza virus infections. It takes three to six months to create a vaccine for a newly emerged virus strain [2]. Under this circumstance, antiviral drug for controlling virus infection is of great importance and necessity in the lag phase of the vaccine manufacturing [4].

The envelope of influenza A viruses contains three important components: ion channel protein M2, surface glycoprotein hemagglutinin (HA) and neuraminidase (NA). The M2 proton channel is responsible for proton transfer which is a required process in viral replication. HA helps the virus recognize and invade the host cell, and NA which functions by cleaving the terminal sialic residues on the host cells can facilitate virus shedding [5,6]. Currently, several types of inhibitors are available to treat this infectious disease, such as M2 inhibitors and NA inhibitors [7,8]. However, numerous drug resistant cases to M2 inhibitors have been reported, so application of the M2 inhibitors was limited during some epidemics [8,9]. To date, four anti-NA drugs have been approved, namely, Oseltamivir, Zanamivir (ZMR), Peramivir, and Laninamivir [10,11,12,13].

NA was divided into two groups based on phylogenetic distinction, group-1 NAs (N1, N4, N5, N8) and group-2 NAs (N2, N3, N6, N7, N9) [14]. Historically, the NA inhibitors were developed by structure-based drug design, exclusively based on group-2 NAs [15]. Different from the group-2 NAs, an additional pocket located adjacent to the conserved active site was first discovered in the *apo* form of N1 in 2006, and this pocket was named as 150-cavity because it is capped by the 150-loop (residues from 147 to 152). Moreover, the 150-cavity in N1 would disappear when a ligand bound in the active site under certain crystallization condition, indicating a slow conformational change of the 150-loop [16]. The conformational change of the 150-loop in group-1 NAs suggests new opportunities for antiviral drug design. In addition, computational solvent mapping and *in silico* screening studies identified the 150-loop and the nearby 430-loop (residues from 429 to 433) are novel druggable hotspot regions [17,18]. Researchers in computational and experimental fields have put a lot of effort in studying the dynamic behaviors of the 150-loop [19,20,21,22,23] and exploring novel inhibitors specifically targeting to this region [24,25,26,27].

Molecular dynamics (MD) simulations have shown that the 150-loop is flexible and can form an extensive open 150-cavity in group-1 NAs [19,20]. Further crystallographic studies have shown that group-1 NAs do have an open 150-cavity [21]. Interestingly, one group’s resolution of a crystal structure of NA of 2009 pandemic influenza (09N1) lacks this 150-cavity [28]. Nevertheless, it was later found that the 150-loop was still able to exhibit an open conformation in 09N1 through experiment and simulations [22,24,29]. This common characteristic of group-1 NAs provides a new opportunity for drug discovery. Several compounds that target the 150-cavity of group-1 NAs proposed by *in silico* methods have been reported [27,30]. In addition, a sialic acid derivative, 3-(p-tolyl) allyl-Neu5Ac2en (ETT, abbreviation reported in PDB 3O9K), was resolved in a crystal complex structure with a hydrophobic side group pointing to the 150-cavity [24]. However, the new derivative has a much lower binding affinity than ZMR, indicating the significant challenges to discover novel high-affinity inhibitors specifically targeting the 150-cavity [24].

**Figure 1:**
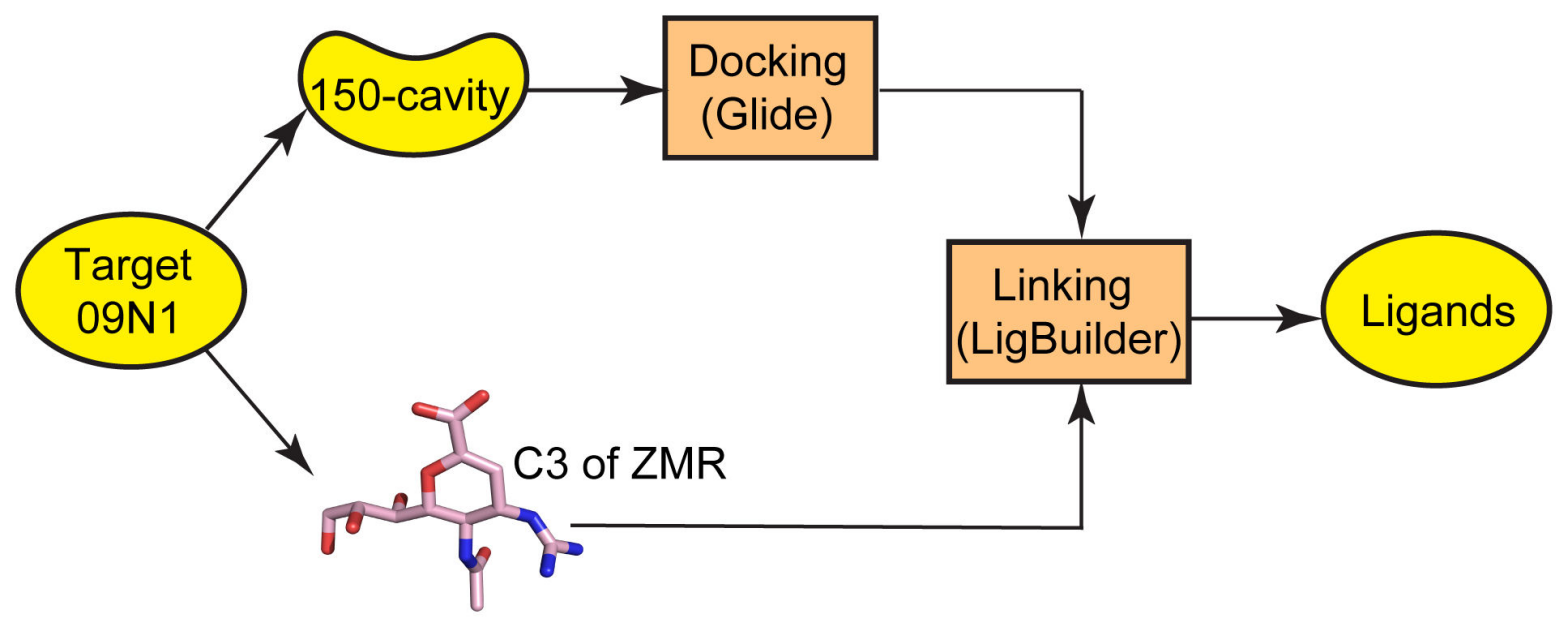
Workflow of the combined method. Workflow of the combined method for selecting drug-like candidates which target the 150-cavity of 09N1. First, selecting fragment candidates through virtual screening based on the structure of 150-cavity in 09N1. Second, linking fragment candidate and scaffold ZMR through C-3 position of ZMR by LigBuilder software. Finally, the successfully linked molecules were tested through MD simulations to testify the stability of the molecules in the binding pocket of 09N1.

In this study we put forth efforts to design such novel inhibitors. A combination of multiple theoretical methods, such as fragment library screening, molecular linking/building and molecular dynamics simulations (Figure 1) were applied to construct and validate new inhibitors and their binding with NA.

## Methods

### Fragment library screening and linking methods

The modeled 09N1 structure with an open 150-loop was prepared as the receptor using Maestro. All crystal water molecules with a distance greater than 5 Å from the protein were deleted, hydrogen atoms were added, and bond orders were assigned. Grids were calculated before docking using the “Receptor Grid Generation” tool in Glide. The grid was built as a cubic box centered at the 150-cavity. The fragment library was composed of 8019 compounds provided by ChemBridge (Divers E set, Chembridge Chemical). Docking was performed using the extra precision mode of Glide [31,32].

**Figure 2:**
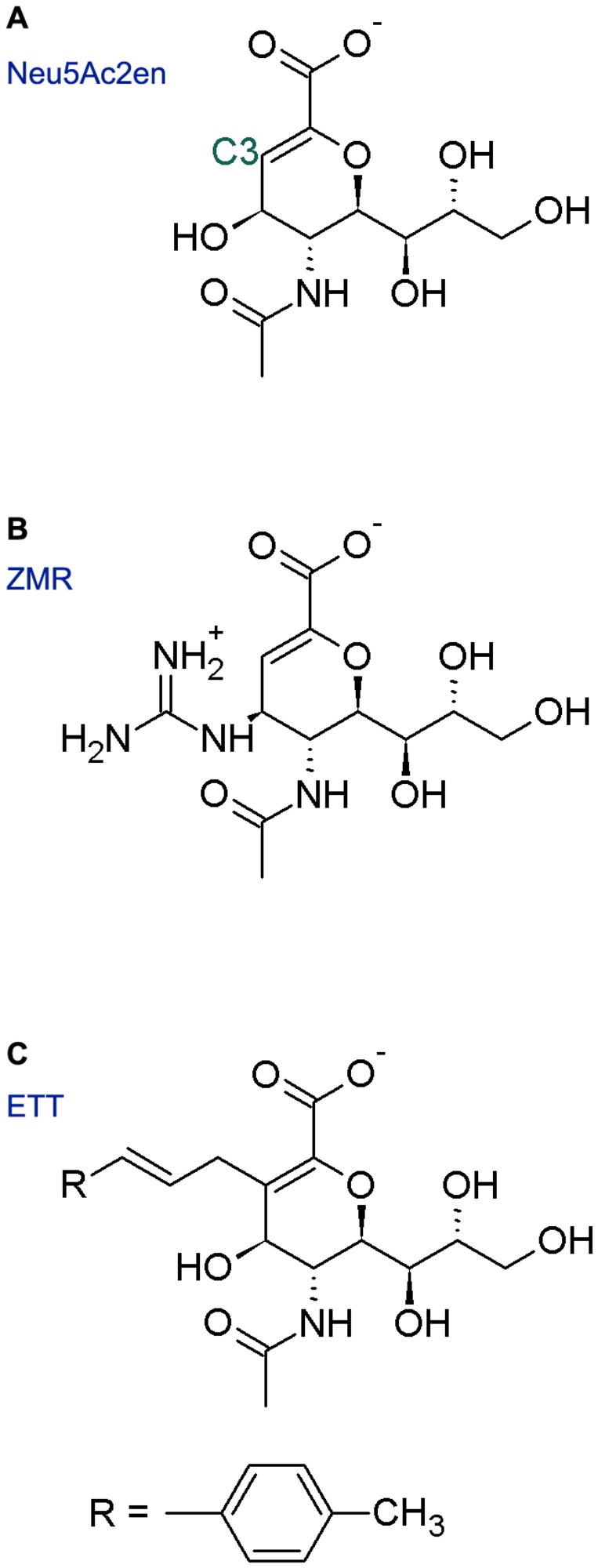
Chemical structures of NA inhibitors and derivatives. Panels A, B and C show the chemical structure of Neu5Ac2en, ZMR and ETT, respectively.

After docking, 30 fragment molecules were found to have glide scores of less than -6 kcal/mol. These fragments were chosen as the fragment candidates to be linked with the scaffold, ZMR drug molecule. The linked molecules were further filtered with a series of criteria: LogP (0-6), molecular weight (300-800 da), number of hydrogen bond donors/receptors (2–10), and binding affinity pK_d_ (5–10). LigBuilder v 1.2 was applied to construct novel inhibitors [33]. In the linking stage, ZMR was used as the linking scaffold onto which the selected fragment candidates were added. The C-3 position on ZMR is the only position that can accommodate growth towards the 150-cavity with minimal distortion of other NA contact sites (Figure 2). In total, 19755 compounds were generated from the linking process, but the majority was filtered using the above criteria. Finally, four ligands were successfully linked. Explicit water MD simulations were performed on NA-ligands complex to verify the stability of the binding modes.

### MD simulation details

Normal MD simulations of ETT, ZMR and the four constructed ligands in complex with N8 and 09N1 were performed. Partial charges of ligands were calculated by Gaussian09 [34] using the R.E.D.-III.4 tool [35]. Atom types were assigned by the antechamber program [36] included in the AMBER10 package [37]. ETT bound with the N8 complex was downloaded from Protein Data Bank with PDB ID: 3O9K [24]. Wild type 09N1 with an open 150-cavity was taken from the crystal structure 3NSS from A/California/04/2009 and modeled using SWISS-MODEL based on the structure of 2HTY from A/Vietnam/1203/4 [16,28,38]. A crystal structure with an open 150-cavity from a 09N1 mutant was recently published [29]. After pair-wise alignment and superimposing all α carbon atoms of the modeled 09N1 structure onto the open state 09N1 crystal structure, an all-atom RMSD value of 0.292 Å was obtained. This small RMSD value indicates that the modeled structure is similar to the crystal structure, so it is a reasonable initial structure for the MD simulations. It is noteworthy that all the structures of NAs were modeled in a functional tetrameric form constructed by Pymol based on the crystal symmetry [39].

Each of these systems was solvated with TIP3P water in an octahedral box [40]. The minimum distance between the protein and the edge of the box was set to 1.0 nm. Sodium and chloride ions were added with a concentration of 100 mM into the system. Protonation states for histidine residues were determined by Chimera software [41]. The GROMACS program suite version 4.5.4 and Amber99SB force field were used in all simulations [42,43]. The simulations were performed in an isothermal-isobaric ensemble (300K, 1bar). Bond length constraints were applied to all bonds that contained hydrogen atoms based on the LINCS protocol [44]. An integration step of 0.002 ps was allowed in simulations. Electrostatic interactions were treated with Particle Mesh Ewald method with a cutoff of 0.9 nm with grid spacing for the FFT grid < 0.12 nm [45]. A cutoff of 1.2 nm was used in the calculation of van der Waals interaction. The details for all the simulation systems can be found in Table 1.

**Table 1:** Detailed information of all the MD simulation systems.

System	No. of atoms	No. of Water (TIP3P)	Time (ns)	Repeated time
ZMR+09N1	161546	46050	20	1
ZMR+N8	156720	44340	20	1
ETT+09N1	161533	46027	20	3
ETT+N8	156671	44305	20	1
Lig 1+09N1	161611	45981	20	1
Lig 2+09N1	161618	45982	20	1
Lig 3+09N1	161624	45984	20	1
Lig 4+09N1	161643	45993	20	1

### Analysis methods

#### Root mean squared deviation (RMSD) calculation

The RMSD of ligand was calculated by superimposing the heavy atoms of the protein onto its initial reference structure. The calculated RMSD represents the overall movement of the ligand relative to the target protein during the simulation.

#### Volume calculation

The volume of the 150-cavity was calculated using POVME program [46]. In the initial structure, a 3D-grid that that covered the 150-cavity (i.e., a crystal PDB structure with an open form of the 150-loop) was created. NA structural snapshots were extracted from the simulation trajectory every 20 ps, and superimposed onto the reference open structure. The previously generated 3D-grid was also superimposed. Grid points overlapping protein atoms in the structural snapshots were systematically deleted. The volume of the 150-cavity was then calculated by a measurement script by counting the remaining grid points. Residues that influenced the volume include all the residues from 147 to 152 in the 150-loop as well as nearby residues coming close to the cavity during the simulations.

#### Binding free energy calculations using MM/PBSA

The binding free energies of all the systems were calculated using a molecular mechanics/Poisson-Boltzmann surface area (MM/PBSA) approach based on 1000 snapshots extracted evenly from the initial 2 ns of single MD trajectory. In MM/PBSA, the binding free energy (∆*G*_bind_) between a ligand and a receptor to form a complex is calculated as

$$\Delta G_{bind}=\Delta H-T\Delta S\approx \Delta E_{MM}+\Delta G_{sol}-T\Delta S$$

$$\Delta E_{MM}=\Delta E_{\mathrm{int}ernal}+\Delta E_{electrostatic}+\Delta E_{vdW}$$

$$\Delta G_{sol}=\Delta G_{PB}+\Delta G_{SA}$$

Where ΔE_MM_, ΔG_sol_ and -T∆S are the changes of the gas phase MM energy, the solvation free energy and the conformational entropy upon binding, respectively. ΔE_MM_ includes ΔE_internal_ (bond, angle and dihedral energies), ΔE_electrostatic_ (electrostatic) and ΔE_vdW_ (van der Waals) energy. ΔG_sol_ is the sum of the electrostatic solvation energy (polar contribution), ΔG_PB_ and the non-electrostatic solvation component (nonpolar contribution) ∆G_SA_. The polar contribution is calculated using PB model, while the nonpolar energy is estimated by solvent accessible surface area (SASA). The conformational entropy change upon binding -T∆S is usually computed by normal-mode analysis on a set of conformational snapshots taken from MD simulations. However, all of these simulations were initiated from a NA structure bound with a ligand, giving the complex a subtle conformational change during the initial 2 ns of simulation. In this study, the -T∆S term is omitted, and we only compare the gas phase non-bonded energy change and solvation energy calculated using MM/PBSA.

#### Force distribution analysis

Force distribution analysis was performed on the 09N1 system bound with ETT and ZMR to track pair-wise force changes during simulations. This analysis was performed using “mdrun” with the rerun option GROMACS software modified by Stacklies et al. [47]. The pair-wise forces between ligands and the active site were monitored at the first 10 ns of the simulation.

## Results and Discussion

### Four ligands identified through docking & linking method

**Figure 3:**
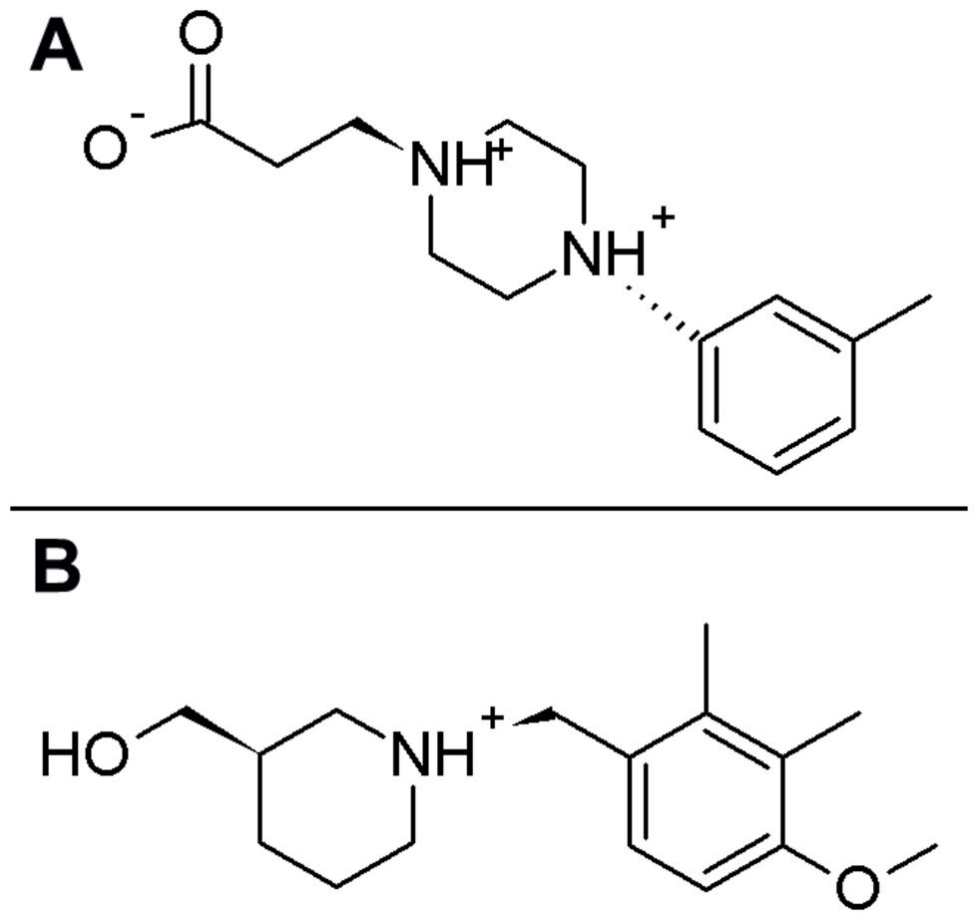
Two-dimensional structures of Fragment 1 and Fragment 8. Two-dimensional structures of Fragment 1 and 8 are shown in panels A and B, respectively.

To identify potential compounds that can be well accommodated in the 150-cavity of NA, we performed *in silico* screening of a fragment compound library (with 8019 members) onto the modeled structure of 09N1, the latest pandemic influenza virus strain. The Glide score algorithm was used to rank members of the fragment library. Thirty fragments had Glide scores less than -6 kcal/mol, and they were selected as fragment candidates for linking. The small molecules were named based on their score ranks with the prefix “Fragment” (Table 2). With the criteria: LogP range 0-6, molecular weight 300-800 Da, number of hydrogen bond in donor/receptor 2-10 and binding affinity pK_d_ 5-10, four compounds were successfully constructed in the linking stage. The linked molecules were prefixed “Lig”. All of them were generated from Fragment 1 and Fragment 8 (Table 3), and the 2-dimensional structures of Fragment 1 and 8 are shown in Figure 3.

**Table 2:** Summary of docking results with docking fragments having a glide score less than -6 kcal/mol.

Entry ID	Chem-Bridge ID	H-acc	H-don	Tot Q	docking score	glide evdw	glide ecoul	glide einternal	XP HBond
1	4023162	3	1	1	-7.37043	-21.4096	-18.254	3.03477	-1.53591
2	9196386	3	2	0	-6.92729	-27.5141	-5.49395	4.676783	-1.20002
3	4023162	3	1	1	-6.81036	-21.0031	-17.3672	1.19626	-1.52328
4	7800757	3	2	1	-6.65618	-16.0363	-12.9647	6.040117	-0.35
5	4032216	3	2	2	-6.64649	-19.3763	-23.0297	0.105478	-1.07038
6	9074551	3	3	1	-6.52798	-22.3908	-15.1795	9.383986	0
7	5281570	3	1	1	-6.46068	-26.0475	-13.9454	1.878679	-1.28173
8	5352152	3	1	1	-6.37494	-24.6968	-13.8561	9.569322	-0.68915
9	9050764	2	1	1	-6.34627	-23.4707	-12.9477	1.065237	-1.43594
10	9128500	2	2	1	-6.32522	-19.5754	-14.8443	4.34129	-0.74179
11	7416480	3	2	1	-6.30067	-25.1667	-13.6941	5.425971	-2.78793
12	5425653	2	1	2	-6.27287	-27.197	-16.659	1.89843	-1.09959
13	4102983	2	2	0	-6.24678	-20.4447	-6.82913	2.515983	-0.34759
14	5423974	2	0	2	-6.24342	-25.9363	-17.5758	2.513569	-1.46303
15	4011111	3	2	1	-6.22181	-13.2892	-16.1706	6.032959	-0.58688
16	4033187	3	1	1	-6.20986	-26.1761	-14.103	1.81429	-0.7
17	5110337	1	2	2	-6.18172	-22.9283	-19.9783	6.062332	0
18	9144090	2	3	1	-6.17131	-24.2924	-14.2719	1.619026	-1.68702
19	5270729	3	0	2	-6.16607	-24.041	-17.2837	11.132024	-0.87137
20	7642164	2	2	2	-6.10683	-28.3099	-13.943	18.058704	-0.66575
21	4004204	3	1	1	-6.09836	-23.3484	-12.2197	1.071324	0
22	9192075	3	2	0	-6.0859	-26.4409	-8.55654	1.233545	-1.90824
23	5410476	2	0	2	-6.07498	-22.6693	-14.3919	7.396954	0
24	5425653	2	1	2	-6.05024	-25.2109	-14.9002	1.216756	-0.9
25	7479241	2	2	0	-6.05011	-27.9463	-7.17434	6.988785	0
26	4039846	3	2	2	-6.0435	-20.7603	-20.261	2.660967	-2.6111
27	9038595	2	1	1	-6.03304	-23.3382	-12.1407	0.301394	-1.87675
28	5181705	3	1	1	-6.03003	-18.6495	-13.0462	3.096679	-1.41122
29	9023327	3	1	2	-6.0057	-26.4847	-18.1268	4.404947	-0.30556
30	5270336	3	1	2	-6.00284	-20.1269	-19.8037	0.235658	-2.5941

**Table 3:** Detailed structures and docking information for Fragment 1 and Fragment 8.

Molecule Name	Entry ID	Chem-Bridge ID	H-acceptor	H-donor	Tot Q	Docking score
Fragment 1	1	4023162	3	1	1	-7.37043
Fragment 8	8	5352152	3	1	1	-6.37494

**Figure 4:**
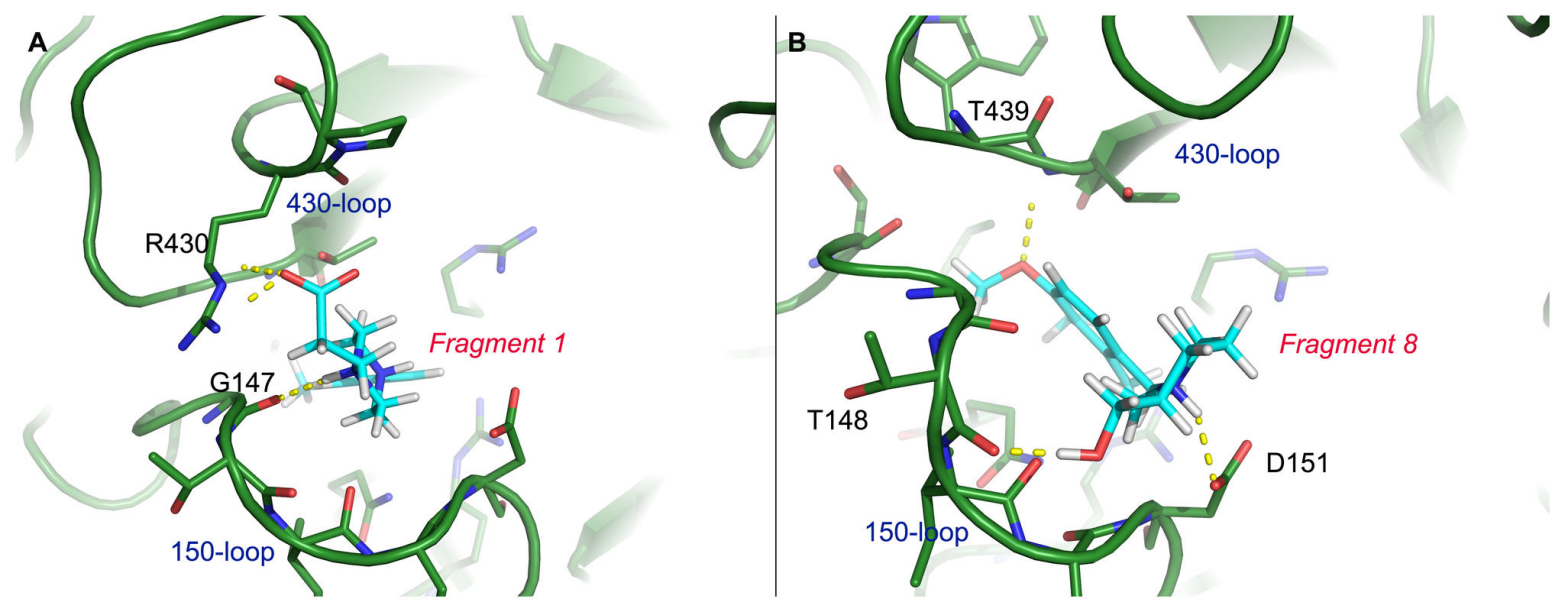
Illustration of virtual screening results of the successfully linked fragments. Panels A and B show the proposed interaction patterns of Fragment 1 and Fragment 8 with 09N1 by virtual screening of a fragment library against the 150-cavity of 09N1. Polar contacts are shown as yellow dashed lines.

Figure 4 shows the proposed interaction patterns between the linked fragments and 09N1. Fragment 1 was proposed to form a hydrogen bond with G147 in the 150-loop and form a salt bridge interaction with R430 in the 430-loop. Moreover, the methylbenzene group was deeply inserted into the 150-cavity, forming a stable hydrophobic contact. For Fragment 8, it forms hydrogen bonds with residues T148 and D151 of the 150-loop and residue T439 of the 430-loop.

**Figure 5:**
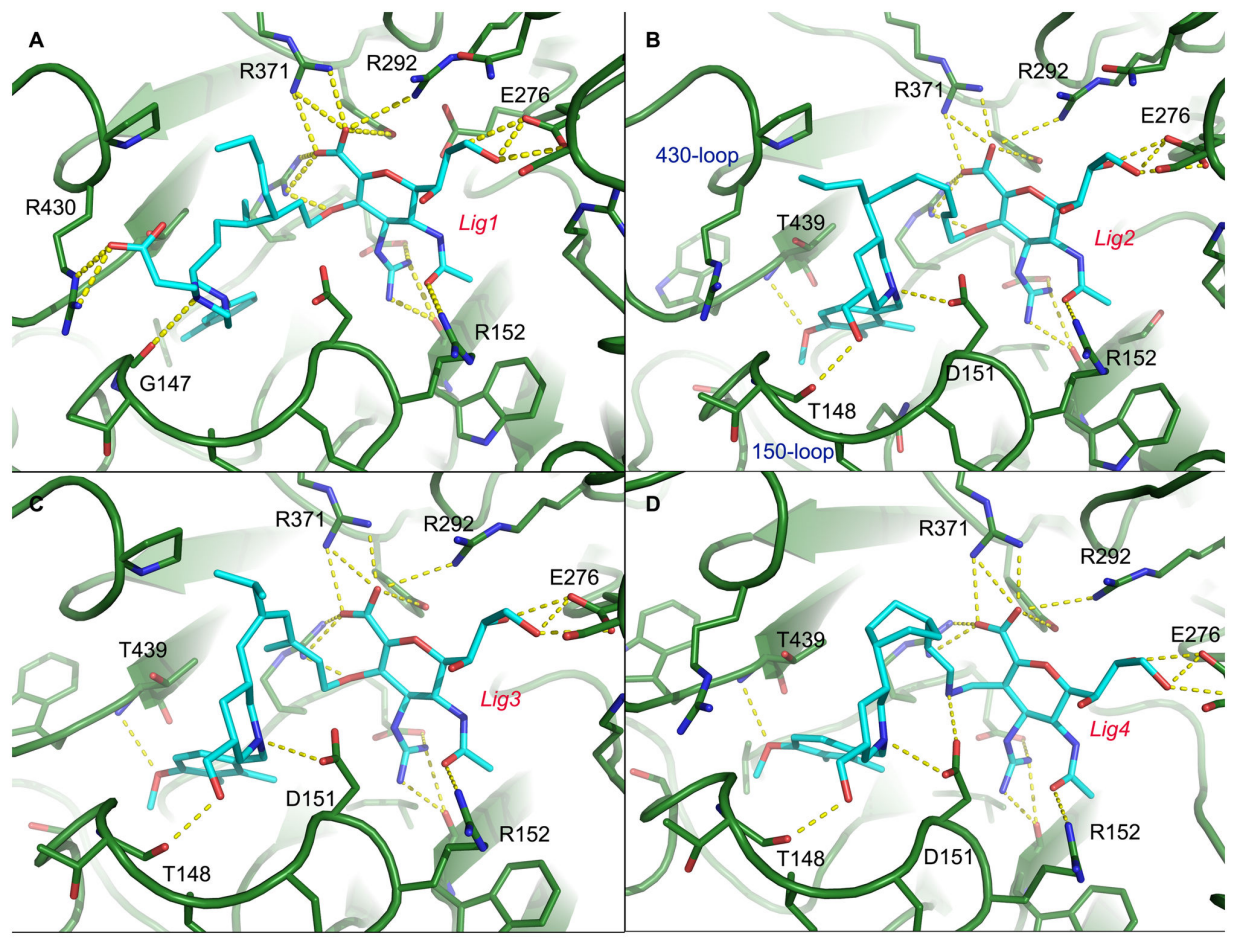
Illustration of the interaction patterns of the linked molecules with 09N1. The proposed binding poses between Lig 1-4 with 09N1 are shown in panels A-D, respectively. All polar contacts are shown as yellow dashed line.

**Figure 6:**
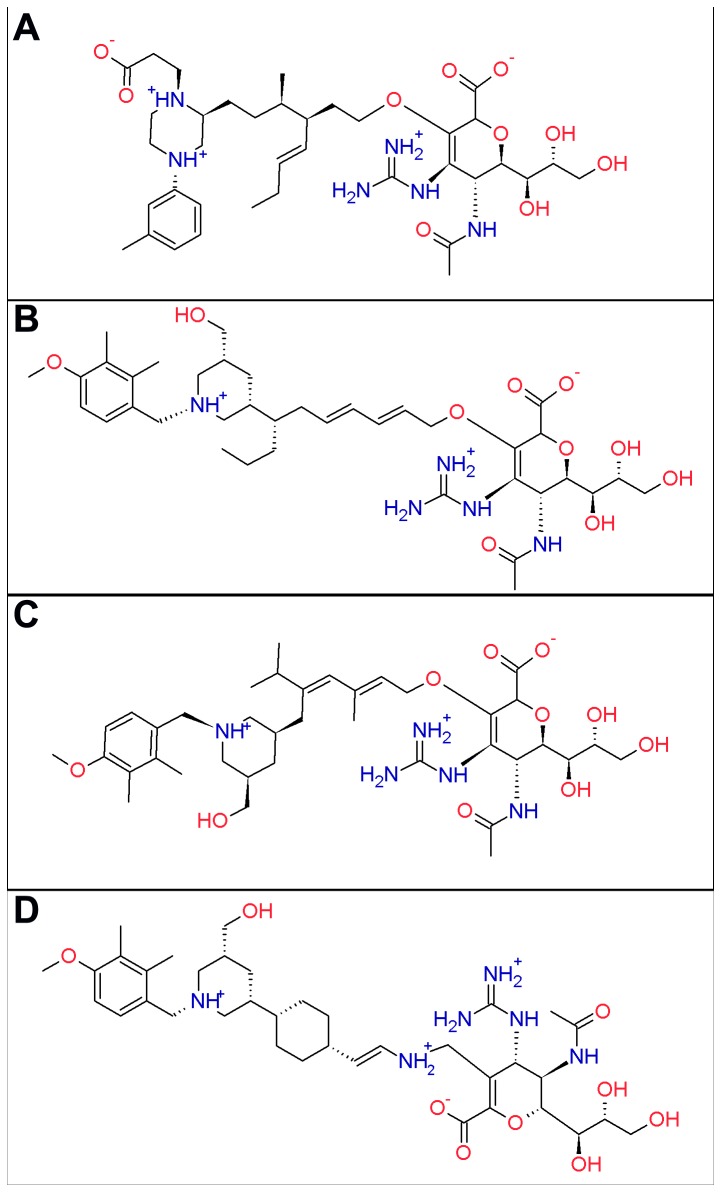
Two-dimensional structures of Lig1-4. Two-dimensional structures of Lig 1-4 are shown in panels A-D, respectively.

The docked fragment candidates, ZMR and the binding pocket were frozen in the linking stage. Different linkers from a linker library provided by LigBuilder v 1.2 [33] were tested to bridge these two molecules together (Figure 5). The 2-dimensional structures and the basic properties of the linked molecules are presented in Figure 6 and Table 4. For Lig 1, it was constructed by linking ZMR and Fragment 1 through an aliphatic chain. Lig 2, 3 and 4 were built on ZMR and Fragment 8 with different linkers. As shown in Table 4, all the compounds have molecule weights greater than 700 Da, and the linkers combining ZMR and the fragment candidates are flexible. Normal MD simulations were performed to verify whether these ligands can stably bind with 09N1.

**Table 4:** Detailed structural information and properties of the four linked molecules.

Name/ properties	Lig 1 (Fragment 1-1)	Lig 2 (Fragment 8-1)	Lig 3 (Fragment 8-2)	Lig 4 (Fragment 8-3)
Formula	C_37_H_59_N_6_O_10_	C_38_H_60_N_5_O_10_	C_38_H_60_N_5_O_10_	C_37_H_60_N_6_O_9_
MW	747	746	746	732
Log P	3.77	4.00	3.17	1.01
pK_d_	5.08	5.22	5.06	5.19

### Lig 1 can stably interact with 09N1 and lock the 150-loop open

**Figure 7:**
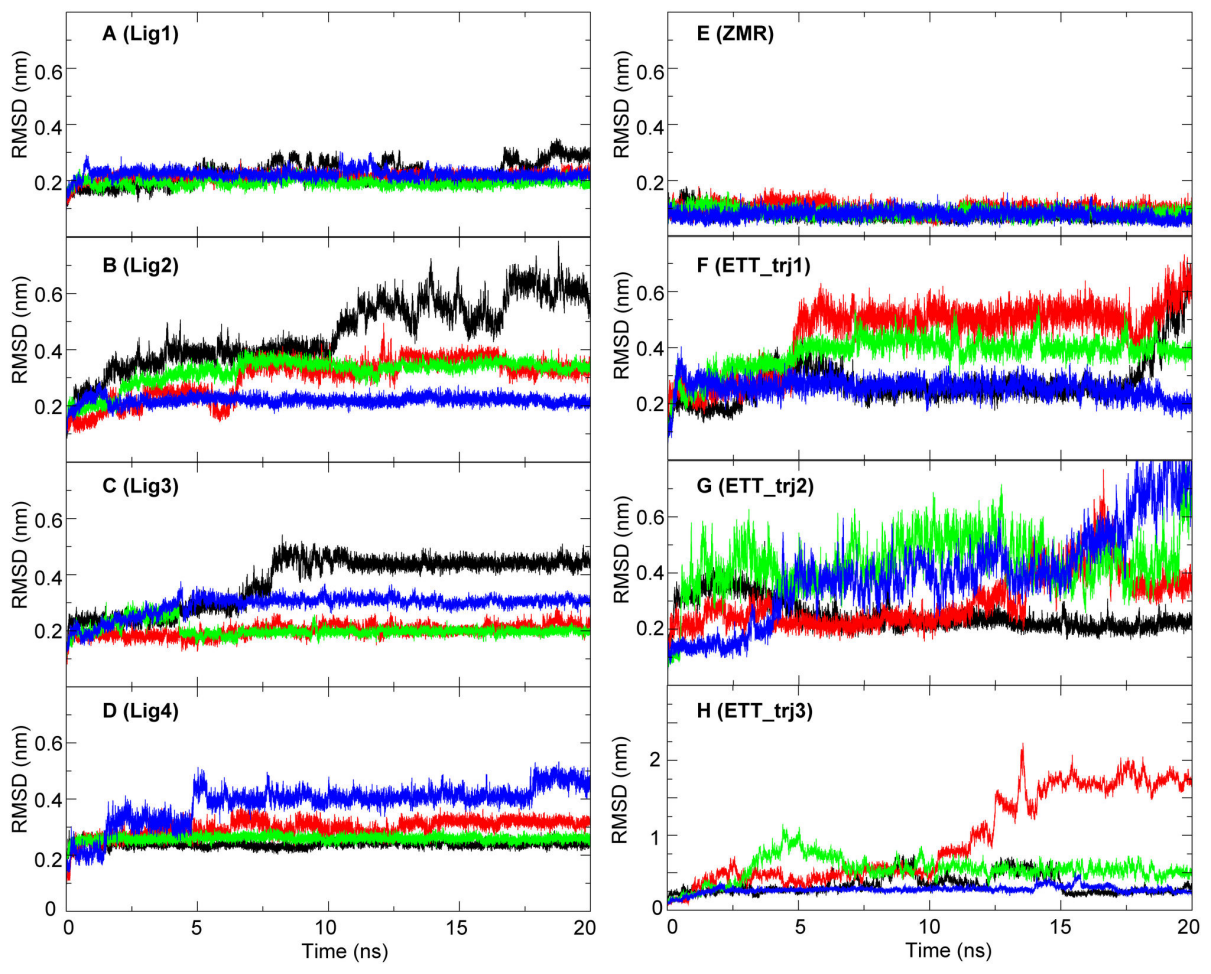
Root Mean Squared Deviation (RMSD) of ligands in different systems. RMSD values of the linked ligands as well as ZMR and ETT bound with 09N1 are shown in panel A-H. Lines colored in black, red, green and blue represented RMSD of protomer A-D in each complex system.

To investigate the stability of ligands in the binding pocket, a series of normal MD simulations were performed. The drug ZMR and the compound ETT, designed by Mark von Itzstein’s group [24], were included in the simulations for comparison. In our setup, each protomer of the tetrameric 09N1 protein was bound with an identical ligand, resulting in four trajectories of the same NA-ligand complex in one simulation. Among the four designed compounds (Figure 7), Lig 1 had the smallest RMSD values, indicating the most stable binding. For the other three ligands, the RMSD values in some protomers were higher and fluctuated around 0.5 nm, representing deviation of the ligands from their proposed binding positions. The large RMSD of the three ligands are displayed as the black curve in Figure 7B and 7C and the blue curve in Figure 7D.

As expected, ZMR remained stable in its binding pocket with a RMSD value less than 0.2 nm. Although ETT was designed to target the 150-cavity, we found that it was unstable in the binding pocket of 09N1. To confirm the instability of ETT, we repeated the simulation three times. In one trajectory, ETT even dissociated from the binding pocket in one protomer (Figure 7H, the red curve). These results indicate that ETT cannot bind stably with 09N1.

**Figure 8:**
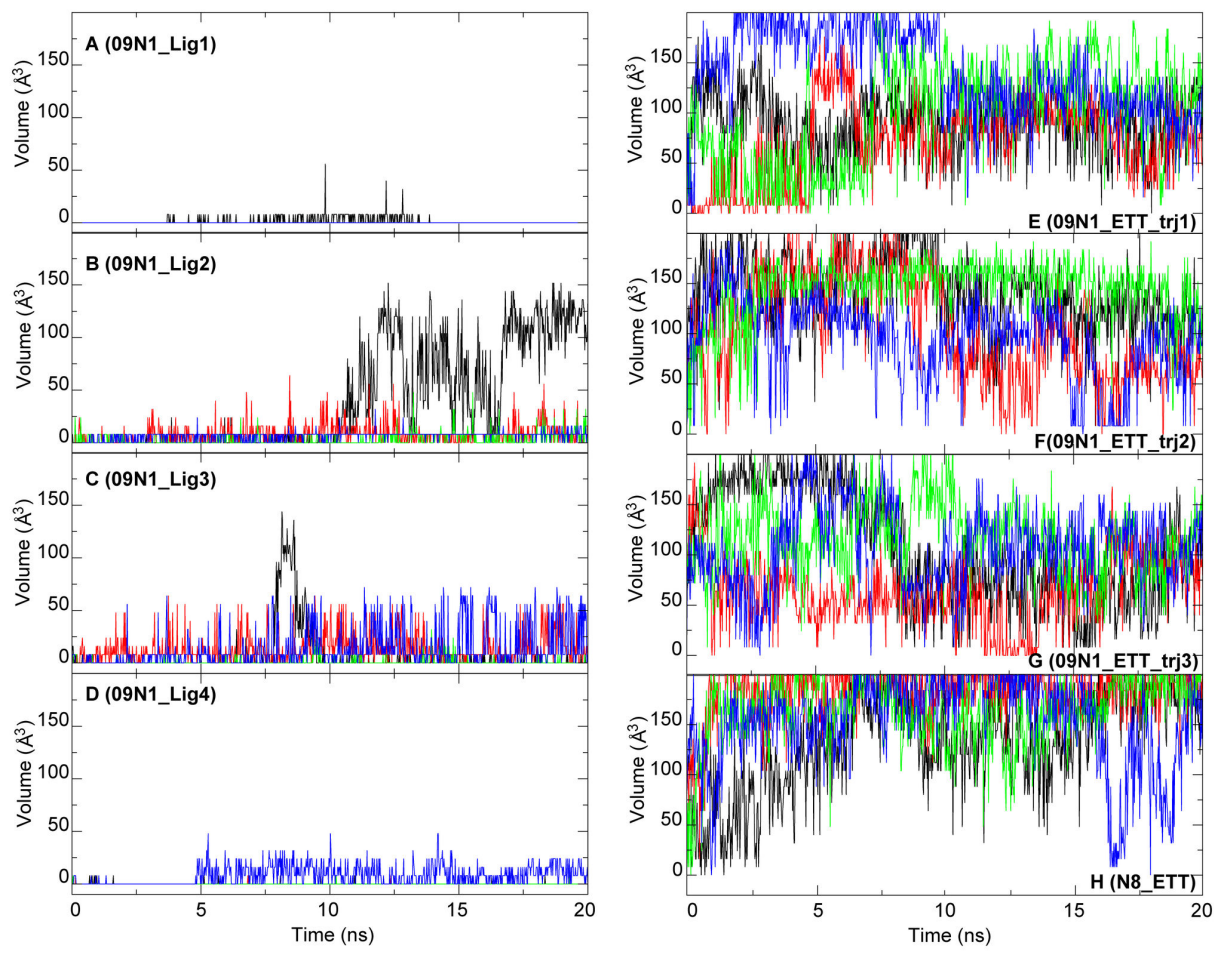
Volumes of the 150-cavity in different systems. The volumes of the 150-cavity in the systems with linked ligands are shown from panel A to D. Simulations of ETT bound in the 09N1 NAs were repeated 3 times, and the volumes of the 150-cavity in these systems are shown from panel E to G. The volume of the 150-cavity in N8 bound with ETT is shown in panel H. Lines colored in black, red, green and blue represented volume of the 150-cavity in protomer A to D for each complex system.

The compounds specifically designed to interact with the 150-loop were expected to fully occupy the 150-cavity during the simulation. Thus the volume of the 150-cavity is a sensitive reaction coordinate for the ligand binding. ETT was designed to lock the 150-loop in an open configuration. The volumes of the 150-cavity of the crystal structure are 8 Å^3^ with the presence of ETT and 112 Å^3^ without ETT. Figure 8 shows the evolution of the volume of the 150-cavity during simulations in different systems. When bound with Lig 1, the volumes of the 150-cavity were zero in three protomers, and fluctuated between 0 and 8 Å^3^ in the last protomer (Figure 8A). Lig 2 and 3 binding showed greater cavity volumes (Figure 8B and 8C). In the presence of Lig 4, the volume of the 150-cavity was maintained below 50 Å^3^ (Figure 8D). Clearly, among the four linked ligands, Lig 1 maintained the 150-cavity at the minimal size, indicating that it can interact most stably with the 150-loop as designed.

On the contrary, simulation data showed that ETT was not able to stay firmly in the cavity in 09N1. The cavity opened quickly from the beginning of the simulations and closed occasionally along the trajectories (Figure 8E–G). We modeled the 09N1 structure with an open 150-cavity and suspected that the largely open 150-cavity may arise from the modeled structure. To eliminate such a possibility, we performed the simulation using the originally resolved complex crystal structure: N8 bound with ETT (PDB ID: 3O9K). The results are shown in Figure 8H. The volumes of the 150-cavity in the four protomers were enlarged and sometimes greater than 200 Å^3^. These results confirmed that ETT does not have the capability to steadily lock the 150-cavity.

### Lig 1 increases its binding affinity by stably interacting with G147, D151, E119 and R430

MM/PBSA was performed to estimate the binding affinity of Lig 1. ZMR and ETT were also included for comparison (Table 5). The value of PB total energy (ΔG_bind_+T∆S), which is the sum of ΔE_MM_ and ΔG_sol_, indicated that Lig 1 had a lower binding energy (-62.68 kcal/mol) compared to ZMR (-35.33 kcal/mol) and ETT (-7.04 kcal/mol), respectively. Gas phase electrostatic interactions and van der Waals interactions (ΔE_electrostatic_ and ΔE_vdW_) made the biggest contributions to binding. Different from ZMR and Lig 1, ETT contains no guanidine group resulting in a larger ΔE_electrostatic_ value. Thus, weak electrostatic interactions may be one reason for weak ETT NA binding. Another reason could be that the derived hydrophobic side chain on the C-3 position on ETT cannot make stable contacts with NA to compensate for the lost favorable electrostatic interactions.

**Table 5:** Binding energies of ligands in different systems calculated by MM/PBSA.

DELTA	ZMR(09N1)	ZMR(N8)	ETT(09N1)	ETT(N8)	Lig1(09N1)
ΔE_electrostatic_	-222.08 (19.27)	-209.86 (20.25)	-90.72 (8.37)	-87.05 (6.89)	-311.80^i^ (15.33)^ii^
ΔE_vdW_	-25.87 (4.73)	-31.50 (4.01)	-36.37 (3.51)	-38.84 (3.23)	-70.71 (4.99)
ΔE_internal_	0.00 (0.00)	0.00 (0.00)	0.00 (0.00)	0.00 (0.00)	0.00 (0.00)
ΔE_MM_	-247.95 (17.13)	-241.36 (19.04)	-127.09 (7.74)	-125.89 (6.74)	-382.50 (14.09)
ΔG_SA_	-5.10 (0.09)	-5.03 (0.10)	-6.37 (0.23)	-6.90 (0.20)	-10.21 (0.17)
ΔG_PB_	217.72 (10.43)	209.84 (13.53)	126.42 (8.20)	124.27 (8.49)	330.03 (11.39)
ΔG_sol_	212.62 (10.44)	204.81 (13.55)	120.05 (8.14)	117.37 (8.43)	319.82 (11.34)
ΔG_bind_+T∆S	-35.33 (10.03)	-36.55 (8.17)	-7.04 (6.19)	-8.52 (5.98)	-62.68 (7.83)

^i^ unit of energy is kcal/mol.

^ii^ The statistical error was estimated on the basis of the deviation between block averages.

**Figure 9:**
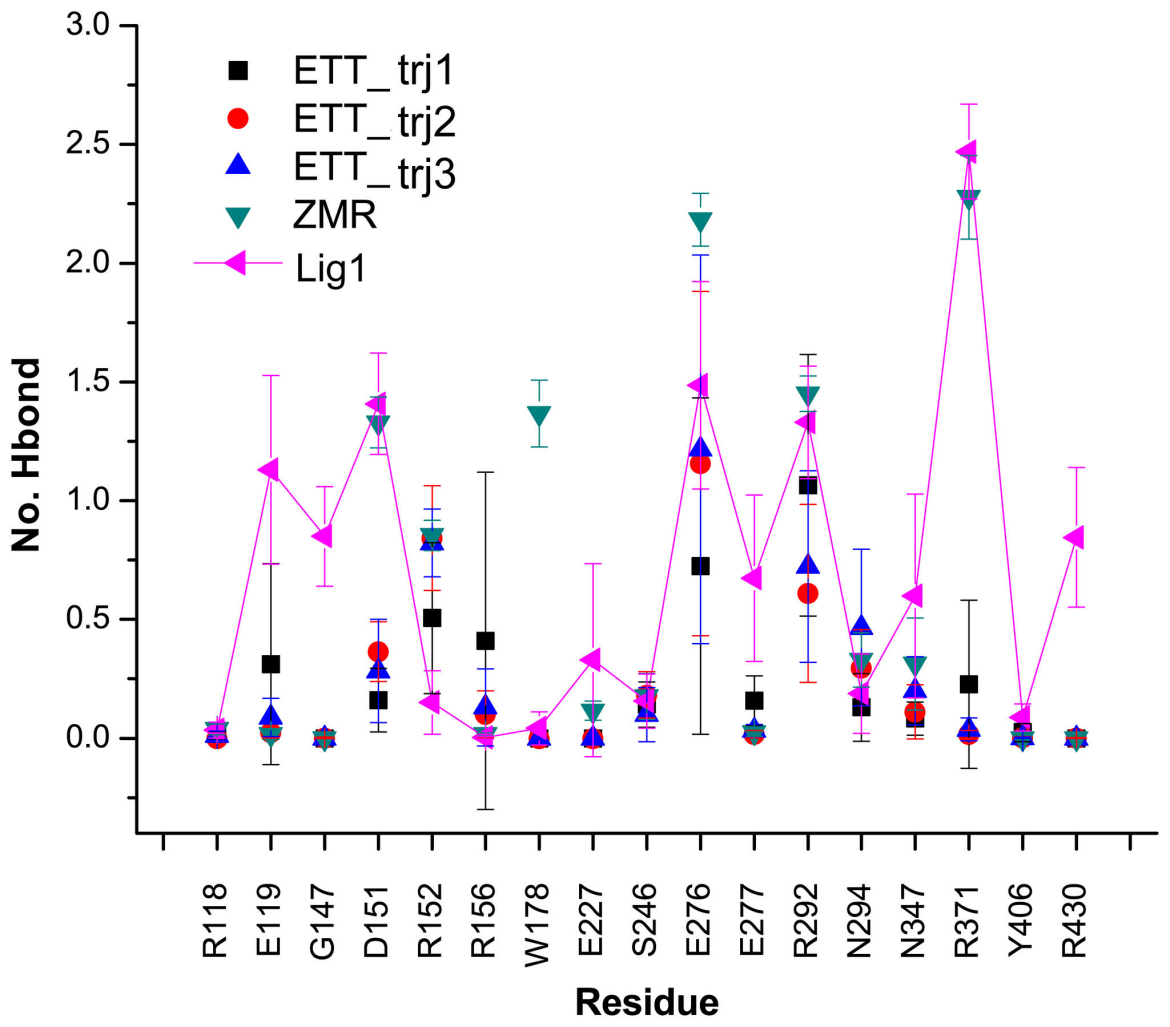
Hydrogen bonds between ligands and active site residues in different systems. The number of hydrogen bonds formed between ligands and active site residues of 09N1. ETT in three repeated trajectories is shown in black, red and blue symbols. ZMR is shown in green color. The number of hydrogen bonds formed between Lig 1 and 09N1 is shown in purple with triangle symbols. Error bars represent standard deviations of the number of hydrogen bonds in four protomers in a single system.

There are several charged residues in the active site of NA. The ligand interacts with the active site mainly through electrostatic interactions or hydrogen bond interactions. To check the detailed interaction between ligands and the active site, hydrogen bond analysis was performed based on the whole trajectories. Figure 9 shows that ETT cannot form stable interactions with 09N1 in three different trajectories. On the contrary, the hydrogen bonds formed between ZMR/Lig 1 and 09N1 remained stable. Compared to ZMR, Lig 1 formed more hydrogen bonds with E119, G147, D151 and R430. Meanwhile the flexibility of Lig 1 is larger than ZMR in the binding pocket of 09N1. G147 and D151 are in the 150-loop and R430 is in the 430-loop. These residues are the original designed targets for Lig 1. The stable hydrogen bonds between Lig 1 and the 150/430-loop indicate that the originally expected interactions were well maintained during simulations.

**Figure 10:**
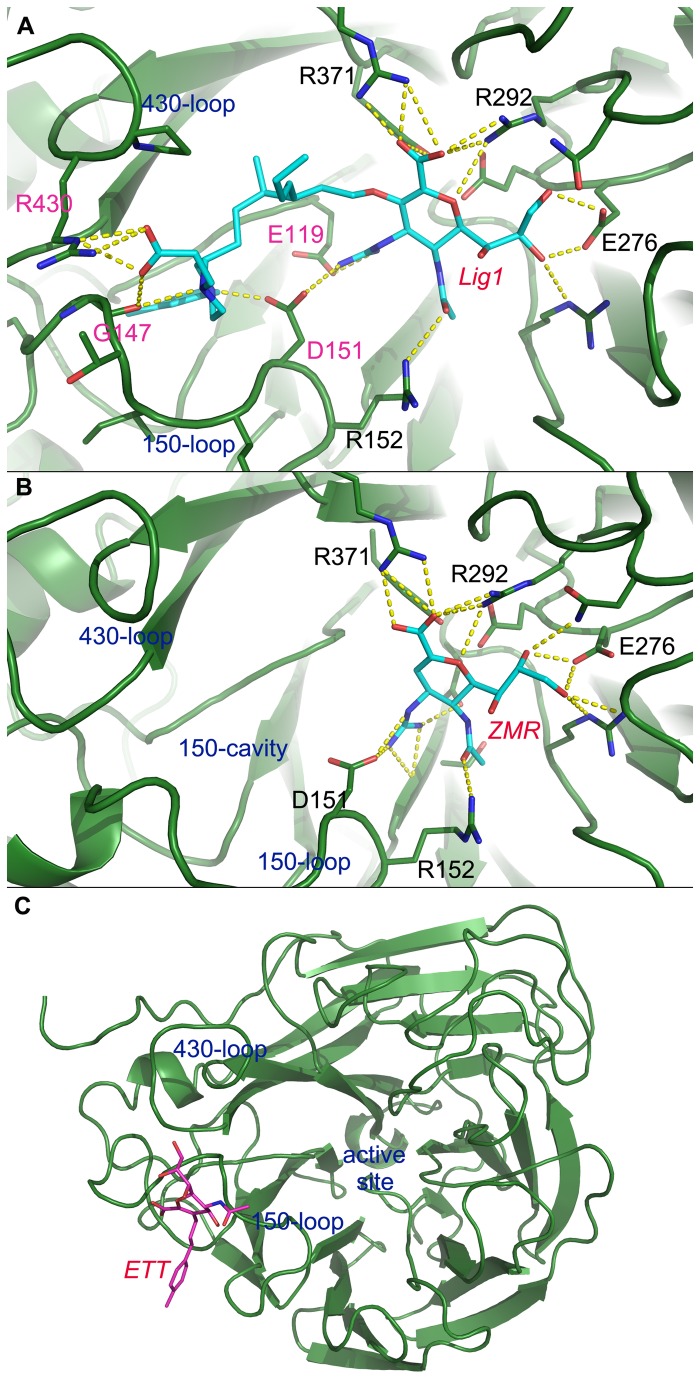
Final snapshot of the complex structure in different systems. Lig 1, ZMR and ETT bound with 09N1 at t=20 ns are shown in panels A, B and C, respectively.

Figure 10 shows the binding modes between ligands and 09N1 of the final snapshot in different systems. ZMR can stably interact with 09N1, with an obviously open 150-cavity on the left side. For Lig 1, most of the interactions between the ZMR base and 09N1 remained, and more polar interactions with the 150-loop and 430-loop formed. The residues that formed additional interactions with Lig 1 are labeled in pink in Figure 10A. However, ETT, which was designed to lock the 150-loop in an open conformation, cannot remain stable in the active site. This is consistent with experimental observations that the inhibition efficiency of ETT towards influenza virus was at micro-molar levels [24]. Compared to the drug ZMR which is efficacious at nano-molar levels, the K_i_ of ETT is too high to be stably bound in the active site. This result also reflects the accuracy of our simulation method since it can reproduce binding stability from experiments.

### Force analysis of ETT binding with 09N1

Our simulation data showed that ETT cannot bind stably with 09N1 or N8. It is important to demystify this instability and provide insights into the application of this scaffold modification method. The pair-wise forces between ETT and the active site of 09N1 were monitored during the first 10 ns of simulation. Similar analysis was performed on ZMR as a positive control.

**Figure 11:**
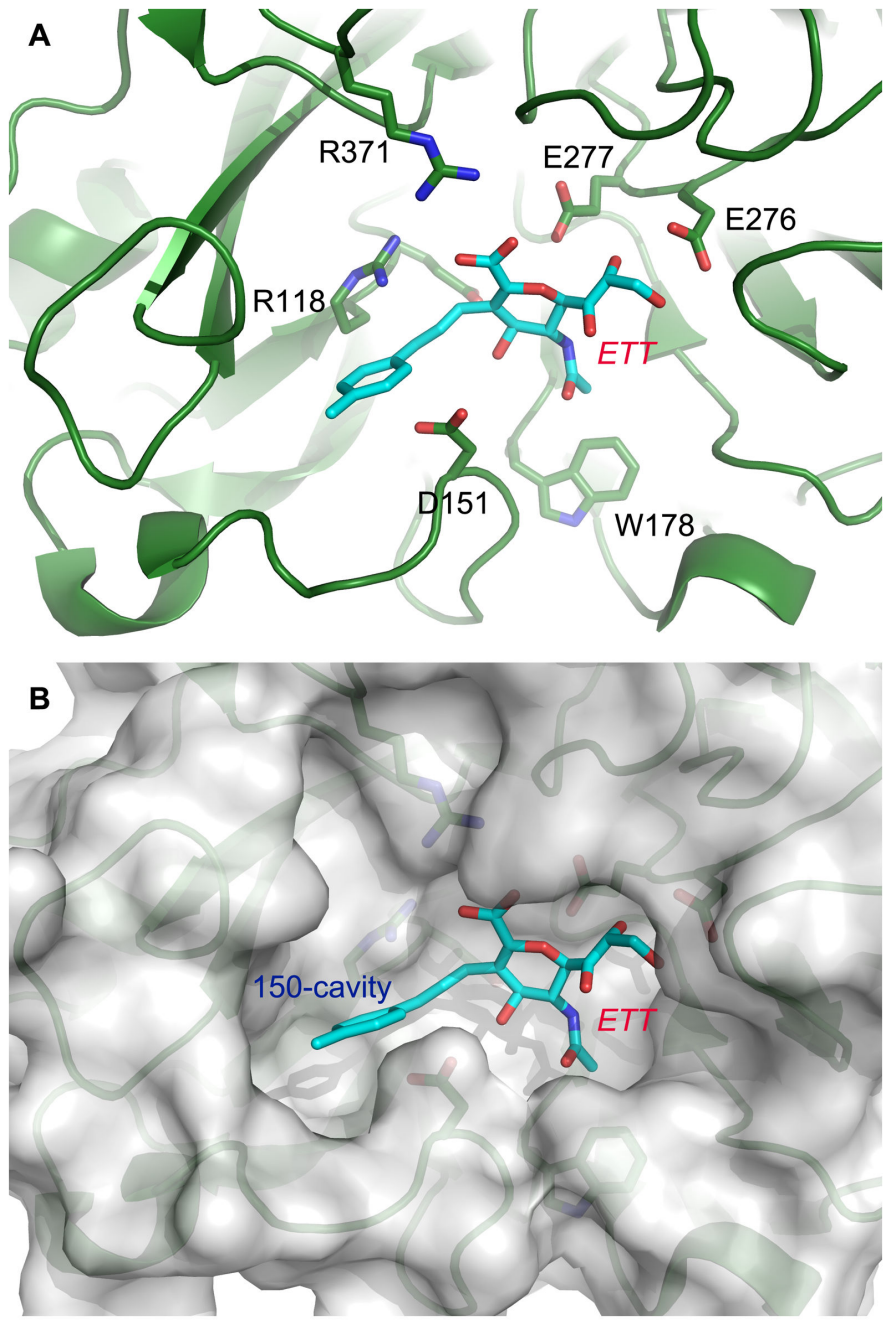
Crystal structures of N8 and ETT complex. The complex is shown in cartoon. ETT is colored in cyan. Nearby active residues are shown in stick representation in panel A. The surface of N8 was calculated for a better illustration of the 150-cavity.

**Figure 12:**
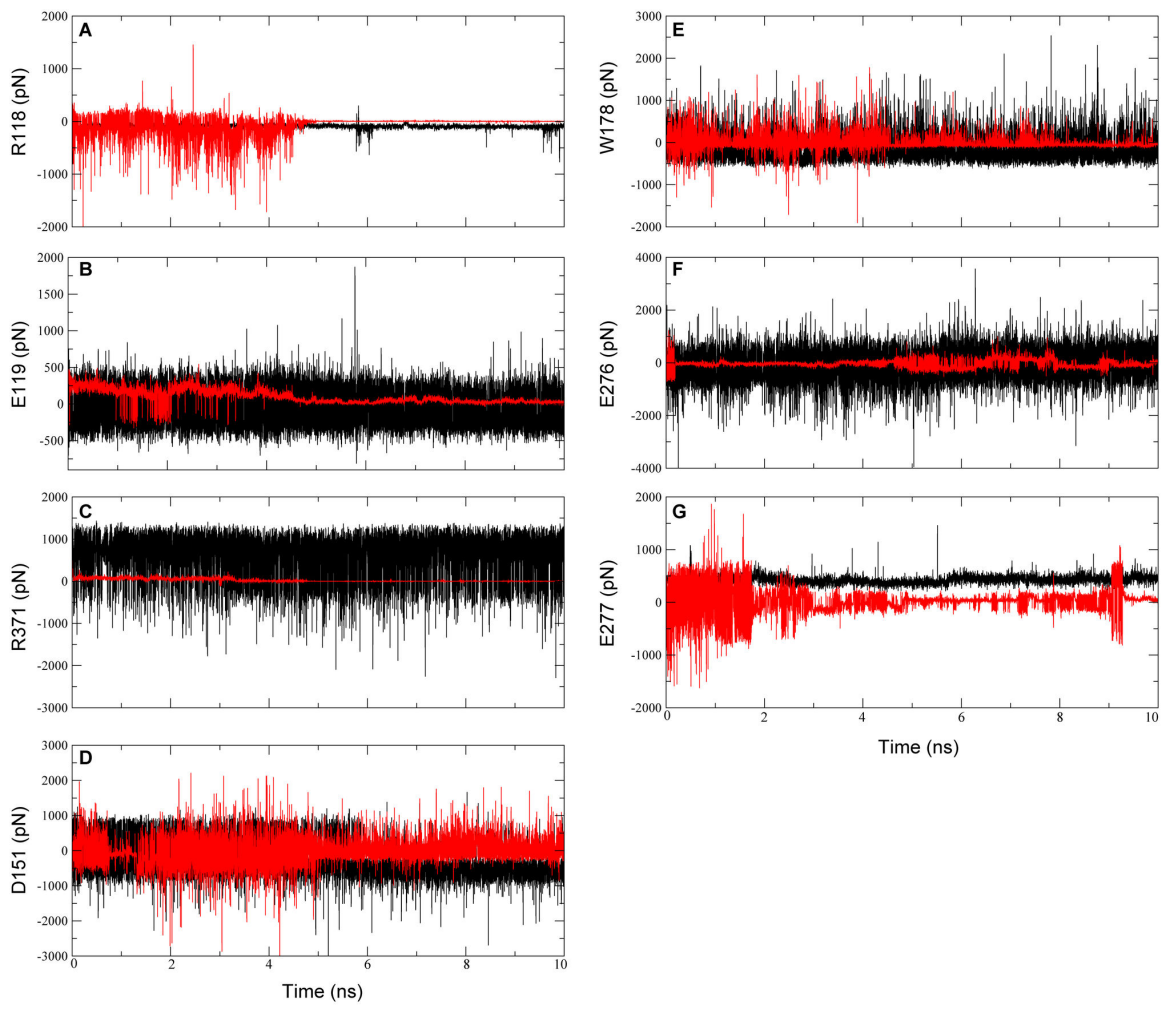
Force distribution analysis between the active site of 09N1 and ETT as well as ZMR. The forces between the active site of 09N1 and ETT are shown in red color curve, and the forces between ZMR and the active site are shown in black curve as a positive control. The pair-wise forces of other protomers and systems can be found in supporting information (Figures S2-S5).

The carboxyl moieties of sialic acid derivatives were originally designed to interact with R118 and R371 in 09N1 (Figure 11A). However, the interaction between R371 and ETT was very weak compared to the interaction between R371 and ZMR in the first 5 ns of simulation (Figure 12C). After 5 ns, this pair-wise force almost vanished, indicating that the distance between ETT and R371 grew to larger than the cutoff value set at the beginning of the simulation to calculate van der Waals or electrostatic forces. Similarly, E119 cannot maintain strong interactions with ETT after 5 ns (Figure 12B). Regarding R118, the pair-wise force formed with ETT at the beginning of the simulation fluctuated, suggesting that the carboxyl group of ETT approached R118. The force disappeared after 5 ns, indicating that ETT moved far away from R118. Clearly, the carboxyl group of ETT cannot maintain its interaction with the active site and moved far away from its original position after a short time of simulation. On the other hand, the force formed between D151 and ETT remained strong, indicating that D151 formed intimate interactions with ETT. Different from ZMR, ETT does not have guanidine group, and there are no polar contacts between ETT and D151. This strong interaction suggested close van der Waals contact between ETT and D151. The minimal distance calculated between ETT and D151 supports our conjecture (Figure S1). At the beginning, the distance fluctuated around 0.4 nm. After 5 ns, this distance decreased to 0.25 nm, indicating that ETT moved towards the direction of the 150-loop. The force formed between E276/E277 and the glycerol group of ETT disappeared around 9 ns, indicating a complete dissociation of ETT from the binding pocket (Figure 12F and 12G). Similarly, the force formed between W178 and ETT was lost after 8 ns (Figure 12E). Based on this force analysis and measurements of the minimal distance between ETT and the active site of 09N1, it is clear that: 1) the carboxyl group of ETT cannot maintain its interactions with R118, E119 and R371 and 2) the newly derived side chain of ETT cannot be stably accommodated in the 150-cavity. These may induce dissociation of ETT from the active site of 09N1.

ETT is a derivative of Neu5Ac2en with difference only on the C-3 position (Figure 2), but cannot stably bind with NA after adding the hydrophobic side group. In the crystal structure, this hydrophobic group points toward the 150-cavity (Figure 11). However, there are no hydrophobic residues inside the 150-cavity in 09N1, so neither hydrophobic contacts nor polar contacts can be formed between ETT and 09N1. In the simulations, ETT was expelled from the binding pocket because of absence of favorable contacts. The above findings suggested that designing intimate contacts between the derived side group and the residues around the 150-loop is of great importance in making efficient sialic acid derivatives.

ZMR was originally designed by replacing the hydroxyl group of Neu5Ac2en with a guanidine group that helped to gain binding affinity through interactions with surrounding acidic residues: the side chains of D151 and E227 and the main chain carbonyls of D151 and W178 [5]. In this study, due to its unique chemical properties, ZMR was chosen as the template. Lig 1 was designed by linking ZMR and the fragment that had the best docking score. Our simulations provided evidence that the intimate interaction between the ZMR part of Lig 1 and the active site of NA was well maintained. Moreover, the designed contacts between the derivative part of Lig 1 and the residues around the 150-loop were also maintained very well. Additionally, the role of the flexible linker in between which allows the whole ligand to stretch in a suitable manner is of indispensable importance. All of these approaches guarantee to design a compound with high binding affinity towards group-1 NAs.

Although the position of the 150-cavity is just beside the binding pocket in group-1 NAs, the main entrance to the 150-cavity is partially blocked by the side chain of D151 (Figure 11B). When designing new derivatives based on sialic acid scaffold, angle and length restrictions have to be considered. For instance, only the C-3 position on sialic acid is suitable for modification to target the 150-cavity. The newly derived side group should be designed to interact with the residues around the 150-cavity in order to simultaneously improve binding affinity and preserve the main scaffold interaction of the ligand. These constraints make it difficult use those methods such as “grow” to build a suitable derivative that meets all the requirements. The protocol that was used in this study to design Lig 1 to target the 150-cavity falls under the category of fragment based ligand design and can be applied to other systems as well.

## Conclusions

The dynamics of the 150-loop have been found to be critical in mediating drug-protein interactions and drug resistance [48,49,50]. The open 150-cavity has become a new target for novel inhibitor design [16,24]. In order to design and verify new ligands that can lock the 150-loop in an open conformation, a combination of multiple computational biology methods, including molecular docking, fragment linking and MD simulations have been applied.

A fragment library was first screened on the 150-cavity, and fragments with extensive interactions with 09N1 based on docking scores were chosen. Then the selected candidates were linked with ZMR using LigBuilder. At the same time, the linked molecules were filtered based on a series of criteria. Finally, all the linked molecules were tested using MD simulations to see whether they could bind stably with the target protein. One ligand has been shown interact stably with 09N1 with high binding affinity.

Extensive simulations were also performed on two additional small molecules, ZMR and ETT. ZMR served as a positive control while ETT was used as a negative control. Our simulation data showed that ZMR stably binds with the receptor. Although ETT was previously proposed to lock the open 150-loop, we showed that ETT actually bound 09N1 with low affinity [24]. In fact, ETT dissociated from 09N1 in MD simulations. By monitoring the pair-wise force formed between ETT and 09N1, the dissociating path was discovered, with the derived hydrophobic group of ETT found to be incapable of maintaining favorable contacts with residues around the 150-loop. Based on these findings, we have concluded that maintaining strong interactions between the newly derived group and the residues around the 150-loop is of great importance in the scaffold modification method.

We hope that this combined method and the newly designed derivatives that lock the 150-loop in an open conformation comprise useful contributions for designing novel inhibitors to combat the spread of influenza virus.

## Supporting Information

Figure S1 **Distance between ETT and the active site of 09N1.** The minimum distance between R371 and carboxyl group of ETT is shown in black. The minimal distance between D151 and ETT is shown in red.
(TIF)

Figure S2 **Pair-wise force between ETT and the active site in all protomers of the first round of simulation.**
(TIF)

Figure S3 **Pair-wise force between ETT and the active site in all protomers of the second round of simulation.**
(TIF)

Figure S4 **Pair-wise force between ETT and the active site in all protomer of the third round of simulation.**
(TIF)

Figure S5 **Pair-wise force between ZMR and the active site in all protomers of the simulation trajectories.**
(TIF)
