# Supplementary figures and images for: Locking the 150-Cavity Open: In Silico Design and Verification of Influenza Neuraminidase Inhibitors
Source: PLoS One. 2013 Aug 27;8(8):e73344. doi: 10.1371/journal.pone.0073344 (PMC3755005; doi:10.1371/journal.pone.0073344)

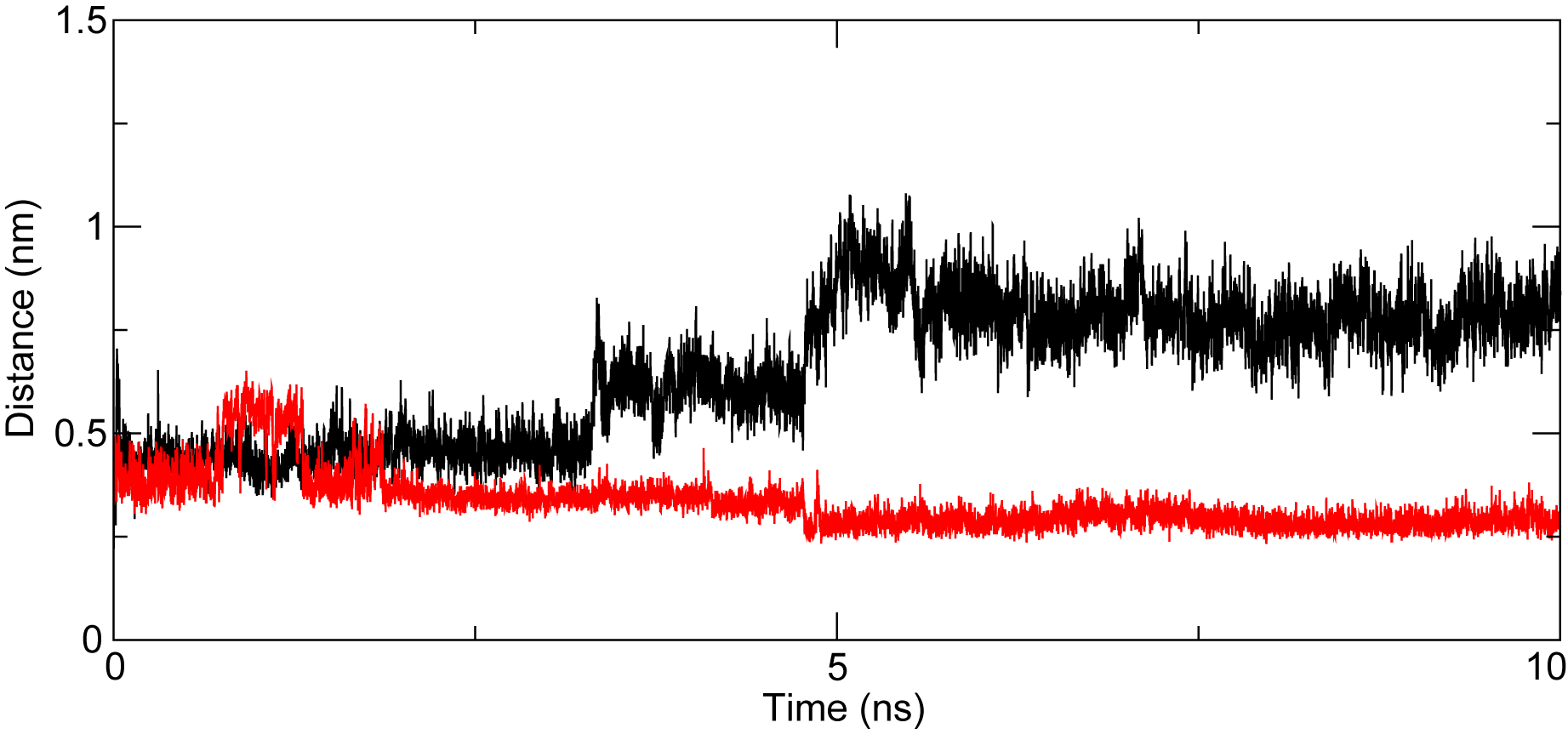

Supplement: Figure S1 — Distance between ETT and the active site of 09N1. The minimum distance between R371 and carboxyl group of ETT is shown in black. The minimal distance between D151 and ETT is shown in red. (TIF) [file pone.0073344.s001.tif]

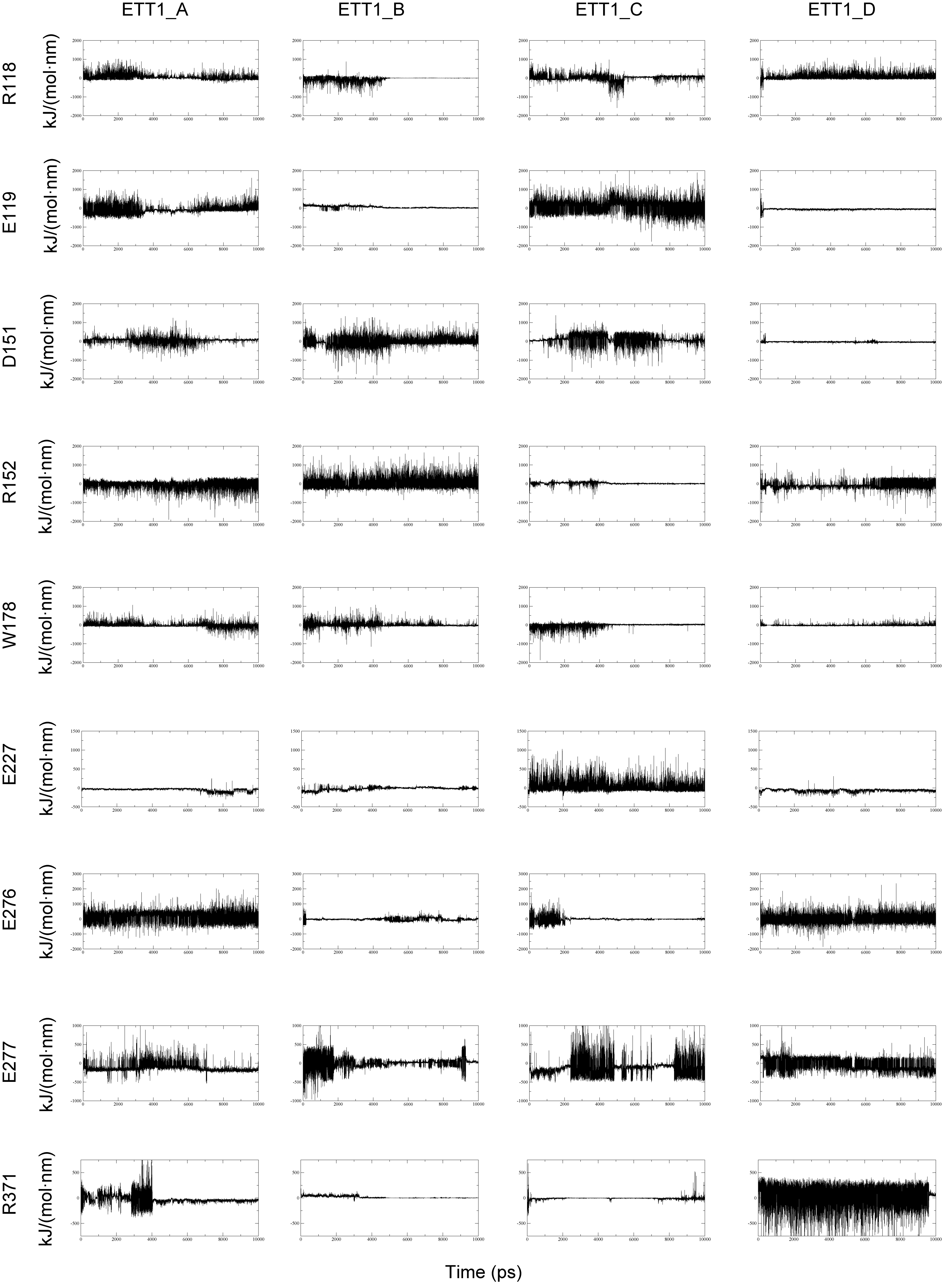

Supplement: Figure S2 — Pair-wise force between ETT and the active site in all protomers of the first round of simulation. (TIF) [file pone.0073344.s002.tif]

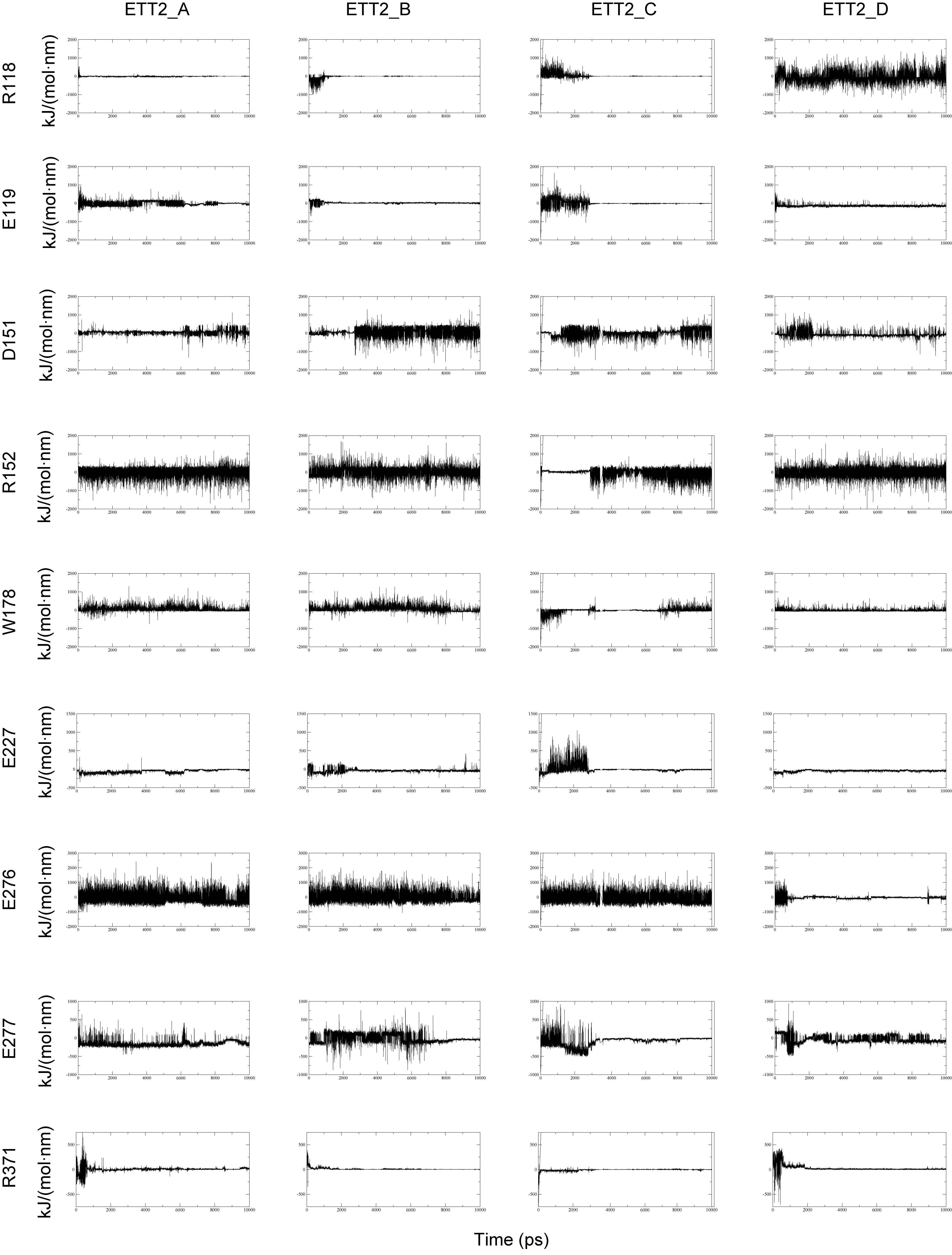

Supplement: Figure S3 — Pair-wise force between ETT and the active site in all protomers of the second round of simulation. (TIF) [file pone.0073344.s003.tif]

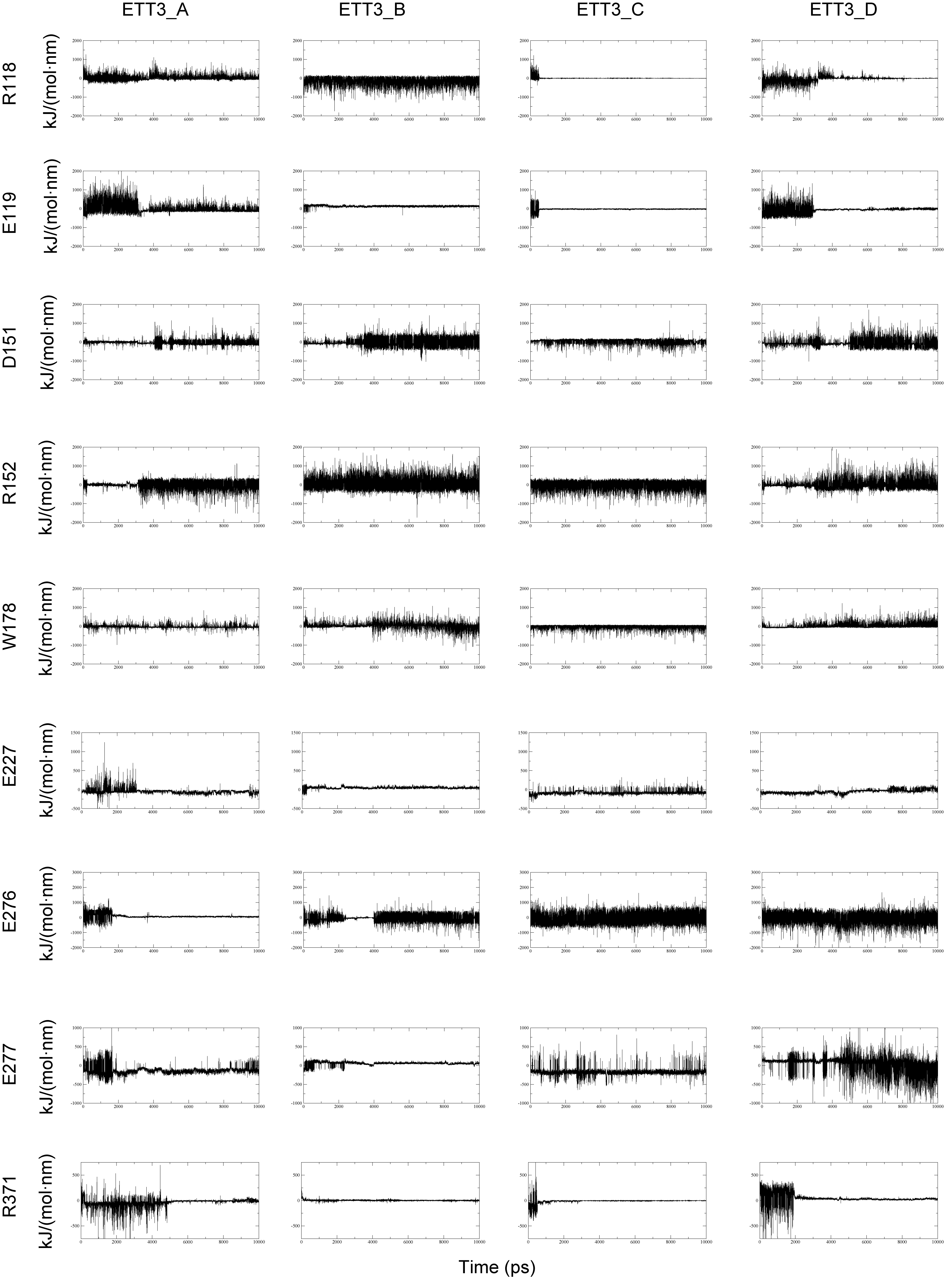

Supplement: Figure S4 — Pair-wise force between ETT and the active site in all protomer of the third round of simulation. (TIF) [file pone.0073344.s004.tif]

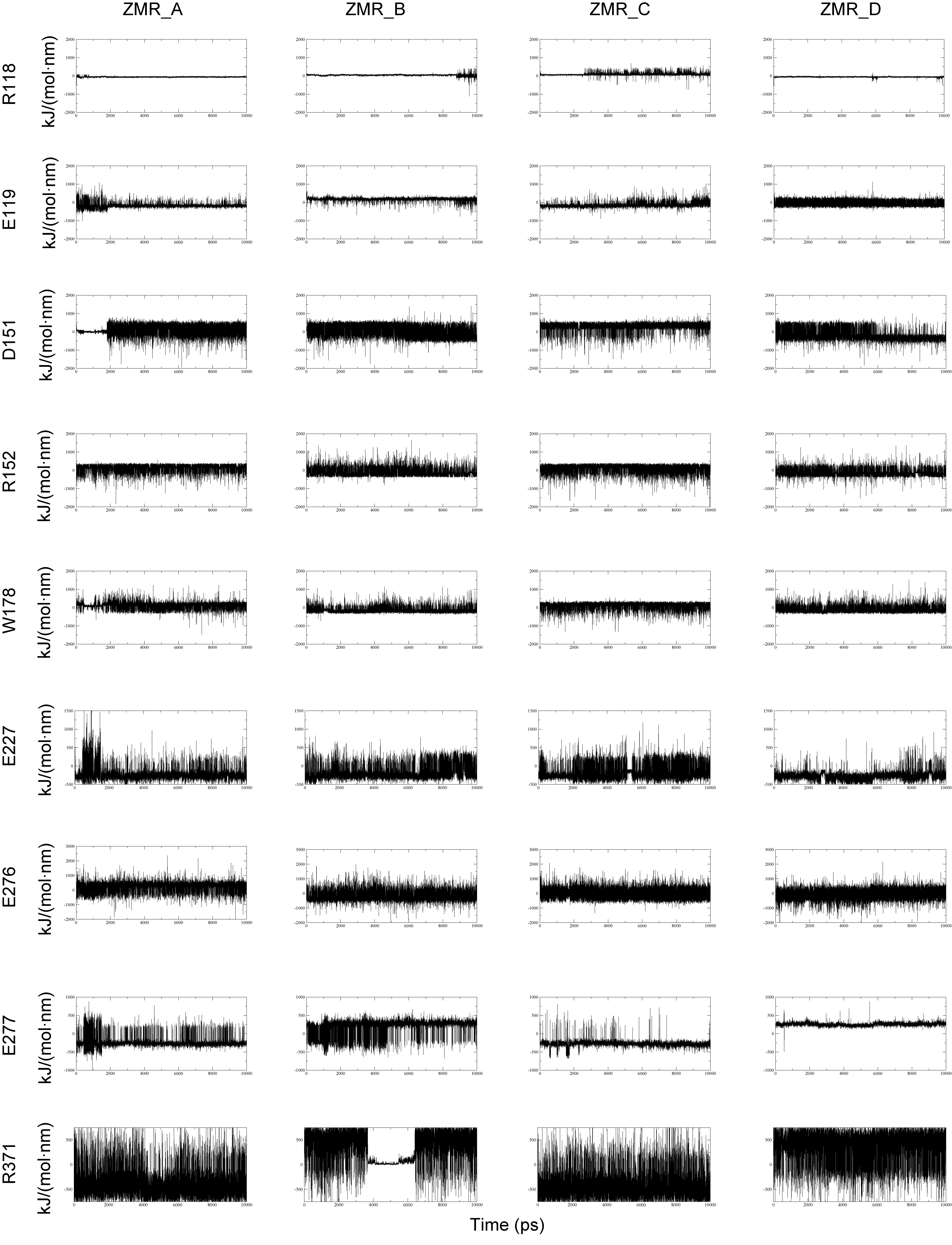

Supplement: Figure S5 — Pair-wise force between ZMR and the active site in all protomers of the simulation trajectories. (TIF) [file pone.0073344.s005.tif]
